# Strength of resting state functional connectivity and local GABA concentrations predict oral reading of real and pseudo-words

**DOI:** 10.1038/s41598-019-47889-9

**Published:** 2019-08-06

**Authors:** Lisa C. Krishnamurthy, Venkatagiri Krishnamurthy, Bruce Crosson, Douglas L. Rothman, Dina M. Schwam, Daphne Greenberg, Kenneth R. Pugh, Robin D. Morris

**Affiliations:** 1 Department of Physics & Astronomy, Georgia State University, Atlanta, GA 30303 United States; 2 Center for Visual and Neurocognitive Rehabilitation, Atlanta VAMC, Decatur, GA 30033 United States; 3 Center for Advanced Brain Imaging, Georgia State University and Georgia Institute of Technology, Atlanta, GA 30318 United States; 4 Department of Neurology, Emory University, Atlanta, GA 30322 United States; 5 Department of Psychology, Georgia State University, Atlanta, GA 30303 United States; 6 Departments of Radiology and Biomedical Imaging, Yale University School of Medicine, New Haven, CT 06520 United States; 7 Department of Biomedical Engineering, Yale University School of Medicine, New Haven, CT 06520 United States; 8 Department of Learning Sciences, Georgia State University, Atlanta, GA 30303 United States; 9 Department of Psychology and Human Services, Mercer University, Macon, GA United States; 10 Haskins Laboratories, New Haven, CT United States; 11 Department of Psychological Sciences, University of Connecticut, Storrs, CT United States

**Keywords:** Predictive markers, Dyslexia

## Abstract

Reading is a learned activity that engages multiple cognitive systems. In a cohort of typical and struggling adult readers we show evidence that successful oral reading of real words is related to gamma-amino-butyric acid (GABA) concentration in the higher-order language system, whereas reading of unfamiliar pseudo-words is not related to GABA in this system. We also demonstrate the capability of resting state functional connectivity (rsFC) combined with GABA measures to predict single real word compared to pseudo-word reading performance. Results show that the strength of rsFC between left fusiform gyrus (L-FG) and higher-order language systems predicts oral reading behavior of real words, irrespective of the local concentration of GABA. On the other hand, pseudo-words, which require grapheme-to-phoneme conversion, are not predicted by the connection between L-FG and higher-order language system. This suggests that L-FG may have a multi-functional role: lexical processing of real words and grapheme-to-phoneme processing of pseudo-words. Additionally, rsFC between L-FG, pre-motor, and putamen areas are positively related to the oral reading of both real and pseudo-words, suggesting that text may be converted into a phoneme sequence for speech initiation and production regardless of whether the stimulus is a real word or pseudo-word. In summary, from a systems neuroscience perspective, we show that: (i) strong rsFC between higher order visual, language, and pre-motor areas can predict and differentiate efficient oral reading of real and pseudo-words. (ii) GABA measures, along with rsFC, help to further differentiate the neural pathways for previously learned real words versus unfamiliar pseudo-words.

## Introduction

Reading is a culturally invented activity that is predominantly and explicitly an acquired higher order cognitive skill requiring complex learning over years of education, exposure, and practice. Research indicates that the reading system is a network built using other existing neurocognitive brain networks, engaging and integrating visuospatial pattern-recognition, language, attention, memory, executive, and motor networks in the process. Atypical connections between or within these networks are thought to disrupt the acquisition of efficient reading skills and gives rise to reading disabilities (RD)^1–7^, including atypical inter-hemispheric^1,6^ and intra-hemispheric^8–10^ connections.

Much of our current knowledge of brain-behavior relationships in reading comes from the task fMRI literature (for a comprehensive review see^11^), and has provided a strong foundation for understanding RD^12^. However, in some scenarios it may be difficult to use traditional reading-task related fMRI to study populations with very limited reading abilities, such as beginning readers, struggling adult readers, or patients with aphasia and alexia. This is because such populations may not be able to easily perform the required fMRI reading task which can induce significant performance related anxiety, and which may introduce additional variability in task performance due to a subject’s use of compensatory strategies^13^.

Alternatively, understanding the MR-based ‘resting’ connectivity profile of the typical/atypical reading network is another viable approach to identify the abnormal and/or weak connections at a systems/network level. It has previously been shown that the reading network can be identified using resting state connectivity analysis in typical adult readers^3^, and that resting connectivity patterns within the reading network differ between adult typical and struggling readers^1,4,6^. In typical adult readers, Koyama *et al*.^3^ showed that important areas of functional interaction within the resting reading network include the left posterior middle temporal gyrus and left inferior frontal gyrus (L-IFG), and that seeding from the putative visual word form area in left fusiform gyrus (L-FG) brought about strong positive connectivity to L-IFG. Moreover, two previous studies on young adult readers by Finn *et al*.^1^ and Schurz *et al*.^6^ showed that the connectivity between L-FG and L-IFG was consistently stronger for adult typical readers than struggling readers. From a clinical standpoint, resting state functional connectivity (rsFC) data provides critical evidence for why the network is not establishing an efficient reading circuit, especially since children and adult readers show different resting network profiles^1^. These previous reports establish the groundwork for the utility of MR-based rsFC measures in examining the resting physiology in typical and struggling readers, which has been used as a biomarker across multiple studies despite the inherent variability in fMRI data that may reduce the detection power of brain differences across cohorts^14–16^.

Although numerous task-fMRI studies have focused on understanding the general reading circuitry, the underlying neurochemistry that supports these networks is less well understood. Previous studies using Magnetic Resonance Spectroscopy (MRS) to non-invasively examine neurometabolite concentrations in local brain regions have identified neurochemical differences between child typical and struggling readers^17,18^. Investigating the bilateral primary visual cortex (V1), Pugh *et al*.^18^ identified that glutamate and choline in V1 is related to reading performance and linguistic measures such as phonology and vocabulary in children, but did not find a relationship between gamma-amino butyric acid (GABA) and reading measures. Investigating the anterior cingulate cortex (ACC), Horowitz-Kraus *et al*.^17^ identified that dyslexic kids have a negative association between measures of executive function and choline, as well as executive function and myo-inositol. These studies are important, and establish the utility of understanding neurochemical differences between typical and disabled readers. To specifically understand the role of learning in reading disability, the underlying neurochemistry of learning must be explored.

It is expected that “learning to read” involves active changes in the GABA system, which is distributed heterogeneously across the human brain^19^. GABA is a critical factor in the acquisition of a new skill, and is involved in long term potentiation (LTP) of the hippocampus^20,21^, nuclei of the midbrain^22^, the cerebellum^23^, and neocortex^24^. LTP is an important form of synaptic plasticity involved in memory and learning, and essentially provides the foundation for changes in functional connections between brain areas^25^. A previous task-based fMRI study has shown that learning aspects within the language system involves the L-IFG, striatum, and insula^26^. However, the role of L-IFG GABA in the acquisition of language and reading is not well understood. To address this gap, this study focused on GABA measurements from anterior reading areas (specifically L-IFG) involved in new learning.

Given that reading starts with a written input, the frontal aspects of the reading system receive complex orthographic-lexical information from aspects of the higher order visual system. To facilitate this “information transfer” from this visual system into the frontal system, the visual-frontal connection must be present, intact, and of sufficient strength to provide a high fidelity information transfer, as indicated by previous rsFC literature of the reading system^1,3,6^. Because of the high premium on the strength of these visual-frontal systems connectivity necessary to perform well in reading, in this study we chose to seed from the L-FG, which processes higher-order visual information, and inspect its connectivity strength with frontal and striatal regions, and then integrate this information with the frontal GABA information to better describe these components of the reading system and related reading behavior. However, since the initial processing of the visual aspects of text may not be dependent on V1 GABA^18^, and based on the potential importance of frontal GABA in new learning and learning disabilities, we chose to measure GABA in L-IFG. A recent related study^27^ associated resting-state functional connectivity to baseline GABA levels in primary motor cortex (M1) areas and showed that transcranial direct current stimulation (tDCS) evoked decreases in GABA concentrations while increasing rsFC within the motor network. This change in rsFC and related GABA concentrations (via tDCS) established the need to further understand the complex relationship between rsFC and GABA measures, and how their manipulation may have therapeutic potential. Thus, these exciting results, and an understanding of the reading network, provided a conceptual and methodological framework for combining frontal GABA MRS with resting-state fMRI (seeded in L-FG) to understand the reading circuitry.

The goal of this study is to describe the resting brain connections and frontal GABA levels that support single word oral reading. It is hypothesized that MR-based measures of resting physiology (rsFC and GABA) have the ability to differentiate the neural pathways involved in the reading of real and pseudo words. To test this hypothesis, we recruited 20 adult subject of varying reading ability, and who could be classified into 10 typical and 10 struggling readers. The groups were matched for age and socio-economic status and did not self-report any issues with attention nor related diagnoses. All twenty subjects underwent behavioral testing and MRI scan that included both resting state fMRI (to ascertain functional connectivity) and GABA MR Spectroscopy in left inferior frontal gyrus (to ascertain frontal GABA and glutamate + glutamine (GLX) levels). The frontal GABA levels and strength of functional connectivity between left fusiform gyrus and left frontal and striatal regions were evaluated in relationship to real and pseudo word reading to better understand the brain networks that support oral reading.

## Neurocognitive Model of Oral Reading

Cognitive models of oral reading have previously been described^28–30^, but an understanding of the systems level neural architecture for oral word reading that explains RD are still being developed. Of special interest are the processes underlying the transfer and transduction of information between higher order visual, language, and pre-motor/motor networks, as oral reading necessarily requires the dynamic interplay of all three systems^31^. Even if each of these three component systems is robust in isolation, it is posited that if the information transfer between these systems is impaired, it is difficult to establish the integrated skills needed to read out loud.

**Figure 1:**
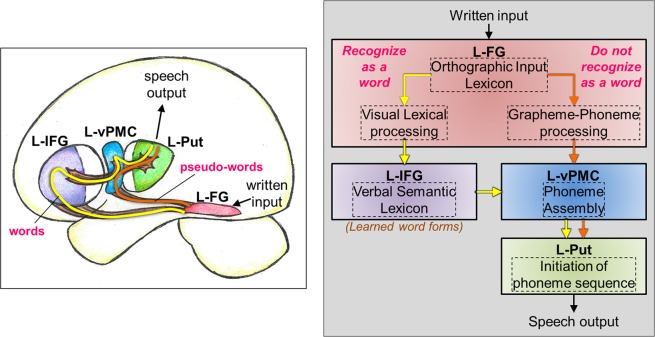
A neurocognitive model of reading out loud displayed graphically on the brain (left) and in box model format (right). The yellow line and arrows represent the processing path if a written input is orally read as a word. The orange line and arrows represent the processing path if a written input is orally read/decoded as a pseudo-word. In the box model diagram, the brain areas are in solid boxes, and the process associated with that brain area is displayed in a dashed box. Note: L-FG: left fusiform gyrus, L-IFG: left inferior frontal gyrus, L-vPMC: left ventral premotor cortex, L-Put: left Putamen.

Figure 1 depicts a representative neurocognitive model of oral reading of words and pseudo-words derived from existing models in the literature^10,32–39^. Starting from the visual system, written input is processed and then transmitted to the left fusiform gyrus (L-FG), wherein the letter sequences are compared to an orthographic input lexicon (i.e., a ‘dictionary’ of language-specific learned letter sequences or orthography) that allows access to lexical output^32^. The L-FG has at least two pathways to further relay the letter sequences, one that is more likely to be upregulated during the processing of real words (while the other one is down regulated), and one that is more likely upregulated for the processing of unfamiliar pseudo-words. This dual-stream characteristic is consistent with recent neurocognitive models of reading^10^ and language^36^. If the letter sequence is recognized as a real word, then the pathway that facilitates visual lexical processing is upregulated (Fig. 1, yellow arrows)^32^. This same pathway can also be upregulated by an unfamiliar letter sequence by incorrectly decoding the pseudo-word as a real word (for example, reading *‘toble*’ as *‘table’*)^39^. On the other hand, if the letter sequence is recognized as a pseudo-word, then the pathway that facilitates grapheme-to-phoneme conversion is upregulated (Fig. 1, orange arrows)^33,34^. For pseudo-words, there is limited need to involve the higher order language (lexical-semantic) processing since the written input does not contain any specific meaning. The general bypassing of the semantic components of the language system has been previously shown for spoken pseudo-words^35^, and we extend this idea to written pseudo-words in this model.

If the written input stimulus is a real word, the information is further routed from L-FG into left inferior frontal gyrus (L-IFG) for additional processing of semantic properties of real words. In the context of the dual-stream language model^36^, it is thought that the dorsal and ventral streams process information in parallel, and converge onto the L-IFG. Within L-IFG, left pars triangularis (L-PTr) is thought to process primarily more semantic information, and left pars opercularis (L-POp) is thought to processes primarily phonological information. The neuroanatomical proximity of L-PTr and L-POp allows for a semantic-phonological processing gradient to exist between the two brain regions in order to facilitate the functional integration of information from both the dorsal (phonological processing) and ventral (semantic processing) streams. It should be noted that irrespective of real or pseudo-words, oral articulation involves phonological coding, such that L-POp is likely involved in phonological processing of both real and pseudo-words^37^.

Following semantic-phonological processing of real words, and phonological processing of pseudo-words, the next step is to assemble sound sequences for the enunciation of real and pseudo-words. It has been shown that left ventral pre-motor cortex is involved in phoneme/sound assembly^37^. Subsequently, the assembled phoneme sequence is compared to the existing phonological output lexicon (dictionary of learned sound sequences), followed by the initiation of the phoneme sequence by the left putamen (L-Put)^38^. The final stage of speech output involves motor planning, selection of competing motor signals, and final motor output to the larynx, tongue, and lips for speech output.

The above model is a derivative of several existing neurocognitive models^10,32–39^, and we believe represents the best available current view of oral reading. More specifically, the model delineated in Fig. 1 has some of the key aspects described in the Dual Route Cascade (DRC) model^40^, but also some components of the Parallel Distributed Processing (PDP) model (also known as the connectionist model or triangle model)^41^. In the proposed study, we evaluated this composite neurocognitive model by using a multi-modal approach of rsFC MRI and GABA and GLX spectroscopy to identify resting brain networks that are associated with oral reading of previously learned (real) words and unfamiliar (pseudo) words. Although the oral reading of both real and pseudo words requires some basic level of learning of the alphabetic principal and phonological recoding, real words require additional higher-level learning of their associated orthographic-lexical features. Unlike task fMRI, resting-state fMRI is a passive task condition where subjects are not engaged in a specific reading task. Thus, identifying the brain areas involved in a cognitive ‘network’ (such as oral reading) during resting condition is a very different challenge from identifying specific brain areas recruited by a given reading task in fMRI.

## Results

### GABA+ and GLX MRS of anterior reading areas in typical and struggling readers

The MRS voxel included left-hemisphere anterior reading areas of L-IFG (both POp and PTr), anterior superior temporal gyrus (L-STG), left ventral pre-motor cortex (L-vPMC), anterior insula (L-Ins), and a small portion of L-Put (Supplementary Fig. 1A). GABA MR Spectra had an average full-width half maximum Cr linewidth of 8.3 ± 3.4 Hz (range of 4.1–14.0 Hz), and LCModel fit Cramer Rao lower bounds (CRLB) of 3.8 ± 1.1% (range of 3–7%) on the GABA moiety at 3.0ppm. The quality of MR spectra were comparable between typical and struggling readers, including no significant difference on the Cr linewidth (p = 0.64) or GABA+ CRLB (p = 0.23). A representative LCModel output of a DIFF and OFF spectra can be seen in Supplementary Fig. 1B. The Creatine normalized GABA+/Cr and GLX/Cr concentrations have a significant reduction with age (Supplementary Fig. 1C, R2 = 0.35 and R^2^ = 0.37 respectively), as expected^42^. The age relationship of the GABA+/Cr and GLX/Cr are removed via covariance analysis for the remainder of the study.

**Figure 2:**
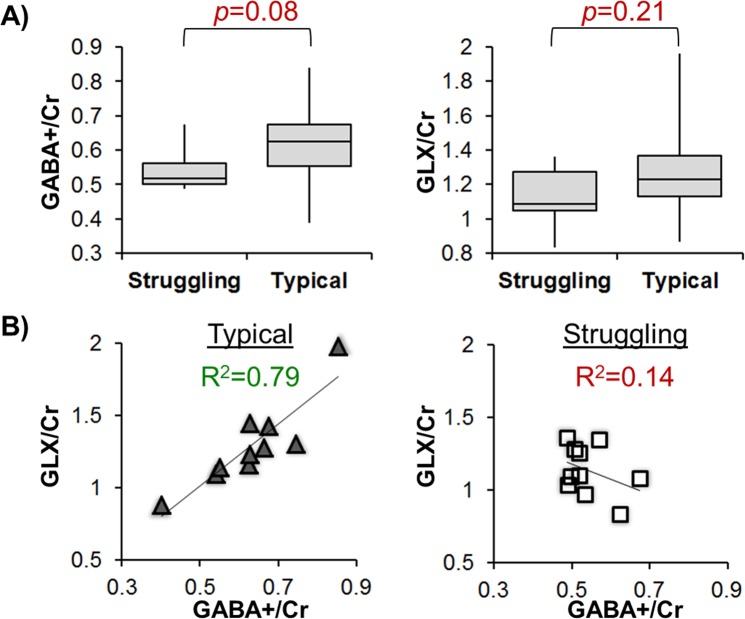
The age-corrected neurotransmitter concentrations for both typical and struggling reader groups. (**A**) Though not significant, the struggling readers have lower GABA+/Cr and GLX/Cr concentrations in the frontal regions. (**B**) The typical readers show a strong relationship between GABA+/Cr and GLX/Cr, whereas struggling readers may have a neurotransmitter imbalance in their frontal system.

The typical and struggling readers did not have a significant difference in either GABA+/Cr or GLX/Cr concentrations when age effects were removed (Fig. 2A), though the struggling readers show a trend in reduced GABA+/Cr compared to typical readers in the frontal regions (p = 0.08). Interestingly, based on linear regression between age-corrected GABA+/Cr and GLX/Cr, the typical readers (Fig. 2B) have a significant positive relationship between GABA+/Cr and GLX/Cr (R2 = 0.79, p = 0.0006) while the struggling readers do not show a significant relationship between these inhibitory and excitatory neurotransmitters (R2 = 0.14, p = 0.29). The difference in GLX/Cr-to-GABA+/Cr slopes between typical and struggling readers is significant, as tested with an interaction effect (p = 0.004).

### rsFC seeded from L-FG

**Figure 3:**
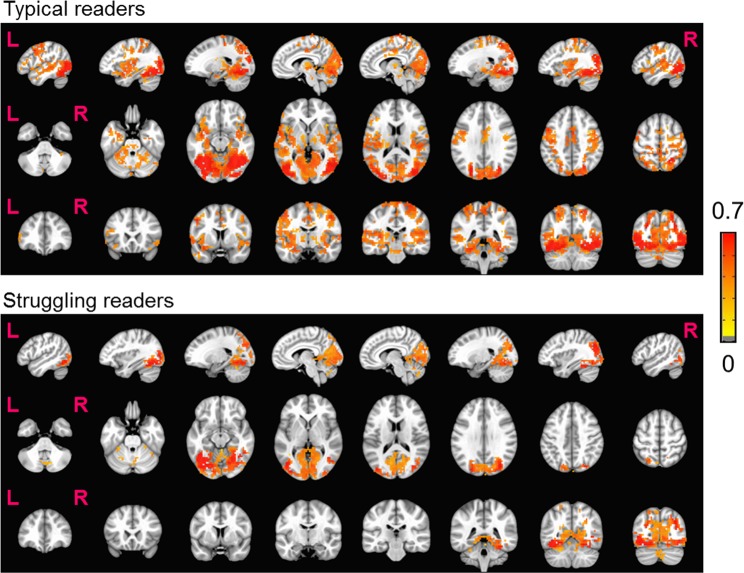
Functional connectivity maps in typical and struggling readers (p = 0.001, cluster size 100, FWE corrected) when seeded from L-FG. Color bar indicates the Z(CC). Note: L = left, R = right.

**Figure 4:**
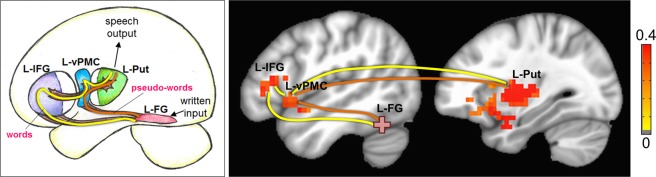
Resting state functional connectivity group difference (Typical - Struggling) results (p = 0.01, cluster size = 30, FWE corrected), displayed with the neurocognitive model of reading. Yellow path represents the path for real words, and the orange path represents the path for pseudo-words. Note: L-FG: left fusiform gyrus, L-IFG: left inferior frontal gyrus, L-vPMC: left ventral pre-motor cortex, L-Put: left putamen. Color bar indicates Z(CC).

Whole-brain rsFC analysis with L-FG as the seed area shows that typical readers have significant connections from L-FG to both language and motor areas, whereas struggling readers do not show significant connections outside of L-FG and associated visual areas (Fig. 3). The group difference results suggest that typical readers have greater connectivity than struggling readers between L-FG and the following brain regions: L-IFG, L-vPMC, and L-Put (Fig. 4).

### GABA-rsFC-behavior relationship (L-FG (seed) to L-IFG connectivity)

**Figure 5:**
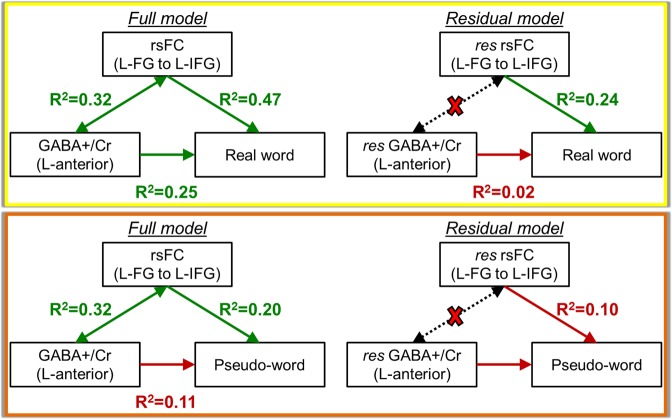
GABA-rsFC-behavior relationships using the anterior GABA+/Cr concentration, rsFC connection strength between L-FG and L-IFG, and reading behavior. The relationship with real words is shown in the yellow box. The relationship with pseudo-words is show in the orange box. The Residual model denotes the relationships when the shared variance between GABA and rsFC is removed, denoted by the dotted arrows and red X.

To further understand the model of oral reading described previously, we integrated the anterior GABA+/Cr measurements, rsFC connection strength (denoted by the Fisher Z-transformed cross correlation coefficient, Z(CC)) between L-FG and L-IFG, and reading behavior measures of real words and pseudo-words for both typical and struggling readers. The relationship of GABA+/Cr, Z(CC), and real word (or pseudo-word) decoding behavior were determined using a multi-step linear regression model. Please see Supplementary Material for details. As seen in Fig. 5, GABA+/Cr level in the anterior reading system has a strong relationship with real word reading (R^2^ = 0.25, p = 0.02), and the L-FG to L-IFG connection strength also exhibits a highly significant relationship with real word reading (R^2^ = 0.47, p = 0.001). When the shared variance between GABA and rsFC is removed in the residual model, the relationship between GABA and the reading of real words is no longer significant (R^2^ = 0.02, p = 0.56), but the residual L-FG to L-IFG connectivity remains a significant correlate of real word reading ability (R^2^ = 0.24, p = 0.03). Further, when inspecting the GABA-rsFC-behavior relationship with pseudo-word reading, it is interesting to note that the GABA+/Cr does not correlate with pseudo-word reading (R^2^ = 0.11, p = 0.16), and the rsFC connection with pseudo-words is no longer significant after the shared variance with GABA+/Cr is removed (R^2^ = 0.10, p = 0.32). Thus, the connection between L-FG and L-IFG is not a predictor for oral decoding of pseudo-words.

### GABA-rsFC-behavior relationship (L-FG (seed) to L-vPMC + L-Put connectivity)

**Figure 6:**
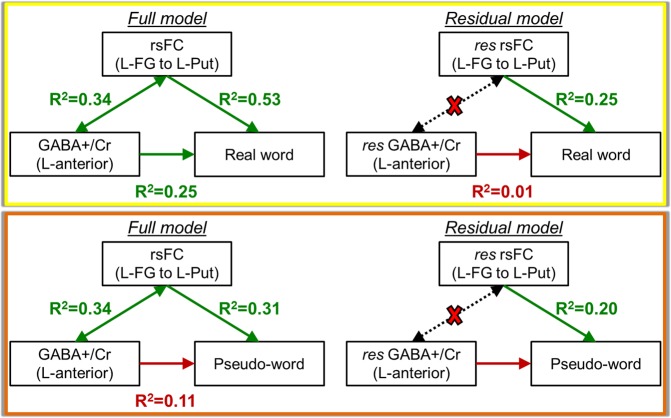
GABA-rsFC-behavior relationships using the anterior GABA+/Cr concentration, rsFC connection strength between L-FG and left pre-motor/motor areas, and reading behavior. The relationship with real words is shown in the yellow box. The relationship with pseudo-words is show in the orange box. The Residual model denotes the relationships when the shared variance between GABA and rsFC is removed, denoted by the dotted arrows and red X.

Similar analysis testing the GABA-rsFC-behavior relationship with real and pseudo-words using all subjects were carried out on the L-FG to pre-motor/motor areas (combined region of interest (ROI) of L-vPMC and L-Put) connection. As seen in Fig. 6, the anterior GABA+/Cr concentration is related to the rsFC connection strength (R^2^ = 0.34, p = 0.01), and both quantities are significantly related to real word reading. When the shared variance between GABA and rsFC is removed, the GABA correlation with word reading is no longer significant, but the connection between L-FG and pre-motor/motor areas continues to significantly describe real word reading (R^2^ = 0.25, p = 0.02). According to the neurocognitive model, the anterior GABA concentration and rsFC between L-FG and left pre-motor/motor areas should describe pseudo-word decoding. After the GABA-rsFC shared variance was removed using the residual model, the correlation between L-FG and L-Put remained as a significant predictor of pseudo-word reading (R^2^ = 0.20, p = 0.05).

## Discussion

In this study we demonstrated the capability of resting physiological measures to predict single word oral reading abilities. The system level resting connectivity profile not only validates a composite neurocognitive model, but also highlights the importance of information transfer (or the lack thereof) in RD. Based on the word type (i.e., real or pseudo-word), the oral reading behavior is strongly predicted by rsFC between higher-order visual, language, and pre-motor/motor systems needed to execute tasks that require oral reading. Of particular note is the presence of at least two processing paths originating in the L-FG, one that is more likely upregulated by real words, and another that is more likely upregulated during the processing of pseudo-words. The processing of real words are better predicted by the connection between the visual (L-FG) and higher order language system (L-IFG). On the other hand, pseudo-words are more likely to upregulate a path that bypasses the higher order language system, and are better predicted by the connection strength between visual (L-FG) and pre-motor/motor system (L-vPMC and L-Put) for speech output.

It is thought that GABA is involved in modulation of learning via synaptogenesis^43^. Our spectroscopy results show that average baseline GABA+/Cr and GLX/Cr concentrations in the anterior portion of the reading network are not significantly different between typical and struggling adult readers, but that GABA concentrations are trending lower in struggling readers compared to typical readers (Fig. 2A, p = 0.08). Though the neurochemistry in the anterior reading system has a relationship with the connectivity profile of L-FG to L-IFG and L-FG to left pre-motor/motor areas, the reading behavior is not correlated with the residual GABA+/Cr concentrations after the shared GABA-rsFC variance is removed (Figs 5 and 6). This suggests that GABA may predict real word reading only if the connections between higher order visual and language areas are present. In contrast, pseudo-words can still be read although they have never been learned as predicted by the composite neurocognitive model (Fig. 1) and observed in our current findings (Fig. 5), and strong functional connectivity between visual recognition mechanisms in L-FG and lexical-semantic mechanisms in L-IFG do not appear important for pseudo-word reading. Overall, these novel findings indicate that GABA in the anterior language system plays an important role in learning to read real words.

Increased glutamate has been implicated in increased neural excitability and neural noise in a reading disability model^18,44,45^. Two studies^18,44^ have previously showed increased glutamate in the mid-line occipital cortices (mOC), which was associated with lower reading skills. Our study did not observe group differences in L-IFG GLX (Fig. 2A, p = 0.21), indicating that the glutamate in L-IFG alone may not be predictive of RD. The different findings in mOC and L-IFG are not contradictory, as regional differences in baseline measures of glutamate^46^ and GABA^47,48^ have previously been observed using MRS. However, the relationship between GLX/Cr and GABA+/Cr was different between typical and struggling readers, indicating that perhaps the excitatory:inhibitory coupling may be of some importance in RD. A tight excitatory:inhibitory coupling is necessary for high precision of neural spiking^44^, which is what we observe in our cohort of typical readers (Fig. 2B, R^2^ = 0.79). As such, the spike timing may be less precise in struggling readers (Fig. 2B, R^2^ = 0.14) due to various neurochemical synthesis and processing issues that need to be further understood.

Although the activation of left fusiform gyrus (L-FG) in reading is well documented, it’s role as predominantly a ‘visual word form area’ (VWFA) was challenged by Hillis *et al*.^32^. In Hillis *et al*.^32^, damage or dysfunction of L-FG due to stroke did not result in written word comprehension deficits, but was associated with deficits in all forms of lexical output suggesting that L-FG is essential for accessing lexical representations in output channels (i.e., oral reading). Our rsFC results for real words show strong connections between L-FG and language (semantic and phonological) and speech systems suggesting that written input of a previously learned form is processed by L-FG to provide access to lexical output (i.e., pathways to process the meaning of the word, sound sequencing and articulation of the word).

When a pseudo-word is processed, then the visual input generally bypasses higher-order language processing, such that the grapheme information is transformed directly to a phonetic representation for output. However, it is unclear whether L-FG plays a role in grapheme-to-phoneme transformation. Dietz *et al*.^33^ suggest that oral reading of unfamiliar/pseudo words is achieved by processing grapheme-to-phoneme conversion rules in the posterior fusiform cortex to sound out a word’s spoken representation, but from a connectionism perspective, these conversions may be better represented by functional connections between L-FG and motor programming areas for speech output (L-vMC and L-Put). Further support for L-FG involvement in grapheme-to-phoneme conversion comes from a lesion study^49^ wherein patients with acquired alexia with L-FG damage showed deficits in grapheme-to-phoneme mapping. Thus, it seems that L-FG (as the foci of our reading network) serves multiple aspects of reading, where connectivity from different anatomical components of L-FG are involved in different cognitive sub-components of reading.

Regardless of whether the written input is a real or pseudo word, the next stage of processing for oral reading involves phonological processing and phoneme assembly. Left pars Opercularis (L-POp) is known for phonological processing^37^, and our rsFC results show a strong connectivity between L-FG and L-POp for typical readers, which is absent in struggling readers (Fig. 3). Since L-POp has been shown to process phonological aspects of sequentially delivered written stimuli^37^, and given that phonological awareness is required to sound out a pseudo-word, we suggest that L-POp plays an important role in phonological encoding of both real and pseudo-words. From the neurochemistry perspective, it is unclear how much of the measured GABA is specific to lexical-semantic processing in L-PTr versus phonological processing in L-POp. Though our GABA voxel of 27 mm^3^ is similar to current standards, the wide coverage of the MRS voxel in the anterior system is unable to capture specific cortical areas of interest (see Supplementary Fig. 1A). This inability to capture specific regions could explain the trending GABA+/Cr differences in the anterior language system between typical and struggling readers (Fig. 2A), since the neurochemistry differences may have arisen from L-PTr rather than other areas included in the MRS voxel.

In terms of phonological assembly, previous studies have shown L-vPMC along with L-POp is associated with reading pseudo-words with atypical orthography^50^. Another study^37^ also showed the involvement of L-vPMC in oral reading. Our rsFC results seeded from L-FG show strong connectivity to ventral portion of L-vPMC irrespective of correlations with real word or pseudo-word reading behavior collected outside of the scanner. Given that L-vPMC is involved in extracting and predicting a sequential pattern for action^51^, but that the sequence does not have to be a learned pattern, connectivity to L-vPMC is meaningful for both real word (learned letter sequence) and pseudo-word (unfamiliar letter sequence). Neuroanatomically, given the proximity of L-vPMC to L-POp, we also suggest that their combined functioning is involved in phonological encoding and assembling sound sequences for oral reading.

Once the sound sequences are assembled, the next step is the initiation of sound sequences for final speech output. A previous study^38^ has shown that both anterior and posterior left putamen are involved in articulating speech with greater activation during overt reading compared to silent reading. More specifically, the anterior putamen was more activated when reading pseudo-words that require initiation of novel sound sequences whereas posterior putamen activated more for oral reading of real words that required learned speech-related movements (of larynx, lips and tongue). Our results show that typical readers have greater functional connectivity between L-FG and left posterior Putamen than struggling readers, and the strength of this connectivity was significantly related to the performance on oral word reading. This suggests that even in resting physiology, the connectivity between L-FG and left posterior putamen predicts the ability to encode speech-related movements for learned words.

The brain-behavior relationships shown in this study were established using resting-state neuroimaging and behavioral reading data that were acquired on separate days. Although one can assume that significant brain processing changes do not typically happen without a significant neurological event over a few days, additional studies should replicate these findings using data from the same day. Since fMRI data has inherent variability^14–16^ arising from day-to-day fluctuations in physiology, it is important to note that these data have previously been shown to be reproducible (ICC > 0.65)^4^. However, it is imperative that the current results are reproduced in a larger cohort since the combination of different modalities (fMRI, MRS, behavior) introduces more complexity in modeling the measurement variability and associated errors. We used typical and struggling adult readers as part of our experimental design to tease apart the underlying learning differences between them that resulted in their current, disparate reading levels, with the assumption that struggling adult readers have had a history of learning disability, and current treatment resistance, in their ability to acquire reading skills. Future work could incorporate more on-line and real time active learning paradigms and specific interventions (combined with acquiring more specific GABA MRS from multiple, relevant systems) to more accurately tease apart the dynamic interplay of active learning and established reading levels. Although the role of visual, language and motor systems in efficient cognitive processing of reading is able to be described in this study, the causal architecture of information flow should be explored in future work using dynamic causal modeling, Granger causality, structural equation modeling, or other techniques capable of detecting direction of information flow during cognitive processing. Finally, our composite neurocognitive model that is described in Fig. 1 is consistent with DRC but could potentially be mapped onto aspects of the PDP model. Given the multiple pathways presumed in our neurocognitive model, the core assumption is that distinct pathways can drive access to lexical output. The computational questions about whether and how semantics might provide support to the orthographic-to-phonologic mapping drives much of the debate between proponents of the DRC versus fully connectionist and interactive PDP models (such as the Division of Labor Model of Seidenberg and colleagues). However, these computational differences can arise in similar “multiple pathway” brain models with variable inter-dependencies amongst these pathways^52^. Future work using reading task-based fMRI connectivity, along with more sophisticated computational approaches, may shed some light on embedded semantic processors using a connectionist approach.

In summary, this study provides the first evidence that resting measures of neurochemistry and functional connectivity can predict reading behavior using a neurocognitive model of oral reading that differentiates real and pseudo-word pathways. More specifically, GABA measures, in conjunction with key brain connections, identified those specific neural pathways that predicted oral reading of real (i.e. learned/familiar) versus pseudo (unfamiliar) words. We also show that irrespective of GABA level in the anterior language systems, the existence of connections between visual, language and pre-motor/motor systems is necessary for efficient information flow to produce skilled reading. Finally, resting physiological measures such as rsFC MRI and GABA spectroscopy can provide meaningful reading network information within populations who have difficulties in performing complicated language or reading fMRI tasks, providing another methodological approach for understanding the nature of the neurobiological differences in such groups.

## Materials and Methods

### General procedures

Twenty adults from low socio-economic backgrounds were recruited from the Adult Literacy Research Center (http://education.gsu.edu/research/research-centers/adult-literacy-research-center/alrc-home/) representing a wide range of reading abilities. A sub-group of these subjects have been described in our previous findings^4^. Informed consent was obtained from all subjects for all experimental procedures approved by the joint Georgia State University and Georgia Institute of Technology Center for Advanced Brain Imaging Institutional Review Board. All methods were performed in accordance with the relevant guidelines and regulations of the IRB.

The study protocol involved three separate sessions. In the first, behavioral testing was administered, including the Woodcock Johnson III (WJ3) used to assess various aspects of reading, including single word reading (Letter Word Identification) and pseudo-word reading (Word Attack). The other two sessions involved MRI scans, of which the second MRI contains the scan data relevant for this study. Within this cohort, we identified two sub-groups based on their reading ability levels: ten typical (mean age = 36 ± 11, age range = 20–53, 4 Male, 6 Female) and ten struggling (mean age = 46 ± 13, age range = 20–60, 4 Male, 6 Female) readers. These two sub-groups were identified based on their WJ3 Basic Standard Scores (age-normed) which fell within the typical or average reader range (WJ3 Basic Standard Score > = 90) or fell in the below average or struggling reader range (WJ3 Basic Standard Score < = 85). This latter group’s reading abilities were all below the 15%ile compared to their age peer norms, and all had significant adult literacy program reading interventions with limited gains, suggesting significant treatment resistance.

### Magnetic resonance imaging (MRI) acquisition

MRI scans were acquired on a Siemens 3T Tim Trio MRI scanner (Erlangen, Germany) using the body coil for radio frequency (RF) transmission and a 12-channel phased-array head coil for RF receiving. The resting state functional connectivity (rsFC) MRI time course was acquired with a Blood Oxygen Level Dependent (BOLD) weighted single shot gradient recalled echo planar imaging (EPI) sequence (FoV = 220 × 220 mm^2^, matrix = 64 × 64, 32 slices, interleaved axial acquisition, slice thickness = 4 mm, TR = 2000 ms, TE = 30 ms, FA = 77°, 147 measurements +3 discards). The subjects were instructed to keep their eyes open and blink at a normal rate, not to fall asleep, to remain motionless, and to remain calm and relaxed while gazing at a white fixation cross on a black background. The subject’s head was comfortably packed using foam pads to minimize motion during and between scans. The subject’s heart rate signals were acquired using a pulse oximeter placed on the subject’s left index finger, and the respiratory cycles were captured with a pneumatic respiratory belt placed around the chest. Both types of physiological data were automatically time synced with the rsFC MRI scan.

A high-resolution T1-weighted anatomical image for spatial normalization to MNI template space was acquired with a T1w-MPRAGE sequence (TR = 2250 ms, TE = 4.18 ms, TI = 900 ms, FA = 9°, isotropic resolution = 1 × 1 × 1 mm^3^). A B0 field map was acquired with a dual echo gradient recalled echo sequence to estimate the amount of EPI distortions in the rsFC MRI images (TR = 488 ms, TE1 = 4.92 ms, TE2 = 7.38 ms, FA = 60°).

### Magnetic resonance spectroscopy (MRS) acquisition

The MRS acquisition utilized a J-editing scheme^53^ with the Mescher-Garwood Point Resolved Spectroscopy (MEGA-PRESS)^54^ sequence to separate the small GABA signals from the rest of the MR spectrum (TR = 2000 ms, TE = 68 ms, acquisition bandwidth = 1200 Hz, acquisition duration = 853 ms, total scan duration = 10 min, water suppression bandwidth = 50 Hz, editing pulse bandwidth = 44 Hz, ON editing pulse = 1.9 ppm, OFF editing pulse = 7.5 ppm, voxel size = 3 × 3 × 3 cm^3^). The voxel was placed in L-IFG without any angulation, targeting the area of the reading circuit where the ventral and dorsal language streams are thought to integrate linguistic information. Placement of the voxel was planned individually for each subject using the T1w-MPRAGE images for anatomic reference. An unsuppressed water (H_2_O) spectrum with matching acquisition parameters was also collected from the same region.

### rsFC MRI pre-processing

The rsFC MRI images were processed as previously described^4^. Briefly, the rsFC MRI time course was corrected for slice-timing and bulk head motion. EPI distortions were corrected using the processed B0 field map. Spatial normalization to MNI template space was performed in conjunction with the T1w-MPRAGE using linear and non-linear transforms. Physiological noise correction was applied to the rsFC time course by detrending the shifted respiratory volume per time (RVT; extracted from time-locked pneumatic belt data) and mean beats per minute (MBPM; extracted from time-locked pulse ox data) vectors together in one nuisance regression^55^ for a combined RVTMBPM correction step. To mitigate partial volume effects, the ventricles were masked from the rsFC MRI datasets. The rsFC MRI time course was then low-pass filtered using a Chebyshev II filter with a cut-off frequency of 0.1 Hz^56^, and spatially smoothed with a 6 mm full-width-half-maximum Gaussian filter.

### MRS pre-processing

The MR spectra were preprocessed with in-house Matlab (Natick, MA) scripts, including phasing of difference spectra, spectral registration of ON and OFF spectra^57^, and subsequent alignment of ON and OFF spectra on the Creatine (Cr) peak. The time course of ON and OFF spectra were averaged separately prior to obtaining the difference spectrum (DIFF = ON-OFF). Then the DIFF and OFF spectra were apodized with a 2 Hz exponential filter to improve signal to noise ratio (SNR). LCModel^58,59^ was used to fit simulated basis sets (created in VESPA; http://scion.duhs.duke.edu/vespa/) to the DIFF and OFF spectra to extract gamma-amino butyric acid with coedited macromolecules (GABA+), glutamate and glutamine (GLX), and Creatine (Cr) concentrations in institutional units, using the unsuppressed H_2_O spectra for normalization. The GABA+ and GLX concentrations were then normalized by Cr since the chemical shift displacement artifact is the same for GABA+ and Cr at 3.0 ppm^60^. A CSF-tissue correction was applied^61^ to the GABA+/Cr ad GLX/Cr ratios to account for tissue volume differences across subjects, and then corrected for aging-related effects.

The difference between typical and struggling GABA+/Cr and GLX/Cr was tested using Student’s t-test. The relationship between GLX/Cr and GABA+/Cr in either typical or struggling readers was tested using linear regression. The difference in GLX/Cr-to-GABA+/Cr slopes between typical and struggling readers was tested using the Group as a condition variable, and testing the interaction between Group*GABA+/Cr to describe GLX/Cr.

### Seed-based rsFC analysis

Due to the importance of L-FG in reading, we chose to interrogate the connectivity of the visual system to the language and motor systems by placing a 5 mm seed in the mid-fusiform gyrus (MNI coordinates (−41.2, −59.2, −14)). The time-course from all voxels represented by this seed were averaged for each subject, and then cross-correlated with all other voxels in the brain. The cross-correlations were then Fisher Z-transformed (denoted as Z(CC)) to allow for the use of parametric statistics. Familywise error (FWE) corrected inferences were obtained through Monte Carlo (MC) simulation of the process of image generation, estimated spatial correlation of voxels, cluster detection thresholds and cluster identification^62^ through the *ClustSim* program implemented in AFNI. This program assumes that the underlying spatial correlation of the second-level analysis residuals is Gaussian, which is consistent with results of recent studies^63,64^. Within-group connectivity maps were assessed using one-sample t-test (voxel-wise corrected p = 0.001, cluster size = 100, FWE corrected) and group difference maps were assessed with a two-sample t-test (p = 0.01, cluster size = 30, FWE corrected).

### rsFC-GABA-behavior relationships

To further understand the neurocognitive model of oral reading, we determined the relationship between GABA+/Cr, rsFC connection strength (Z(CC)), and two different reading tasks (WJ3 Letter Word Identification (real word reading) or WJ3 Word Attack (pseudo-word reading)). The GABA+/Cr values were calculated as described above, including the correction for age-related changes. The rsFC connection strength was averaged on the individual Z(CC) maps using the region of interest (ROI) extracted from the group difference results. The significance of the relationships was determined via the R^2^ metric (e.g. how much variance in the specific reading task is accounted for by rsFC connection strength). Assuming that the rsFC connection strength mediates the relationship between GABA+/Cr and reading, we also evaluated the GABA-rsFC relationship with rsFC (resulting in residual Z(CC)) and GABA+/Cr (resulting in residual GABA+/Cr) using semi-partial correlations. The relationship with reading task performance was then reassessed with the residual Z(CC) and residual GABA+/Cr values.

### Significance statement

This study provides the first evidence that resting measures of neurochemistry and functional connectivity can predict oral reading behavior. Of particular note is the presence of at least two processing paths originating in the L-FG, one that is more likely upregulated for lexical processing of real words, and another that is upregulated by grapheme-to-phoneme processing of pseudo-words. Furthermore, the GABA+/Cr concentration in the L-IFG predicts real word reading behavior, but only if the connections between higher order visual and language areas are present. Irrespective of input (real or pseudo-word), the connection between L-FG and pre-motor/motor systems predicts the oral reading performance, suggesting that both real and pseudo-words undergo sound sequencing and initiation for speech output.

## Supplementary information

Supplementary Information
