# Supplementary material for: Strength of resting state functional connectivity and local GABA concentrations predict oral reading of real and pseudo-words
Source: Sci Rep. 2019 Aug 6;9:11385. doi: 10.1038/s41598-019-47889-9 (PMC6684813; doi:10.1038/s41598-019-47889-9)
Supplement: Supplementary file 1 — Supplementary Information [file 41598_2019_47889_MOESM1_ESM.docx]

**Supplementary Information**

**Strength of resting state functional connectivity and local GABA concentrations predict oral reading of real and pseudo-words**

Lisa C. Krishnamurthy^Ϯ,1,2,3^, Venkatagiri Krishnamurthy^Ϯ,2,3,4^, Bruce Crosson^2,3,4,5^, Douglas L. Rothman^6,7^, Dina M. Schwam^8,9^, Daphne Greenberg^8^, Kenneth R. Pugh^6,10,11^, Robin D. Morris^3,5^

^1^Department of Physics & Astronomy, Georgia State University, Atlanta, GA, 30303 ^2^Center for Visual and Neurocognitive Rehabilitation, Atlanta VAMC, Decatur, GA, 30033, ^3^Center for Advanced Brain Imaging, Georgia State University and Georgia Institute of Technology, Atlanta, GA, 30318, ^4^Department of Neurology, Emory University, Atlanta, GA, 30322, ^5^Department of Psychology, Georgia State University, Atlanta, GA, 30303, ^6^Departments of Radiology and Biomedical Imaging, Yale University School of Medicine, New Haven, CT, 06520, ^7^Department of Biomedical Engineering, Yale University School of Medicine, New Haven, CT, 06520 ^8^Department of Learning Sciences, Georgia State University, Atlanta, GA, 30303, ^9^Department of Psychology and Human Services, Mercer University, Macon, GA, ^10^Haskins Laboratories, New Haven, CT, United States, ^11^Department of Psychological Sciences, University of Connecticut, Storrs, CT, United States

^Ϯ^These authors contributed equally

Supplementary Methods

The relationship between GABA+/Cr, Z(CC), and reading behavior were determined using a multi-step linear regression model, developed in the following steps:

**Step 1:** Find the coefficients (slope and intercept) describing the relationships between GABA+/Cr and Z(CC):

$${{GABA+}/{Cr}}_{measured}=A_{1}+B_{1}\cdot Z\left( CC \right)_{measured}$$

$$Z\left( CC \right)_{measured}=A_{2}+B_{2}\cdot{{GABA+}/{Cr}}_{measured}$$

**Step 2:** Calculate the predicted GABA+/Cr or Z(CC) value based on the coefficients obtained in Step 1:

$${{GABA+}/{Cr}}_{predicted}=A_{1}+B_{1}\cdot Z\left( CC \right)_{measured}$$

$$Z\left( CC \right)_{predicted}=A_{2}+B_{2}\cdot{{GABA+}/{Cr}}_{measured}$$

**Step 3:** Extract the residual GABA+/Cr and Z(CC) by subtracting the predicted value (Step 2) from the measured value, thereby removing the influence of one on the other:

$${{GABA+}/{Cr}}_{residual}={{GABA+}/{Cr}}_{measured}-{{GABA+}/{Cr}}_{predicted}$$

$$Z\left( CC \right)_{residual}=Z\left( CC \right)_{measured}-Z\left( CC \right)_{predicted}$$

**Step 4:** Determine the relationship between residual and behavior:

$$behavior=C+D\cdot{{GABA+}/{Cr}}_{residual}$$

$$behavior=E+F\cdot Z\left( CC \right)_{residual}$$

and compare to the full model relationships:

$$behavior=G+H\cdot{{GABA+}/{Cr}}_{measured}$$

$$behavior=I+J\cdot{Z\left( CC \right)}_{measured}$$

Supplementary Figure 1


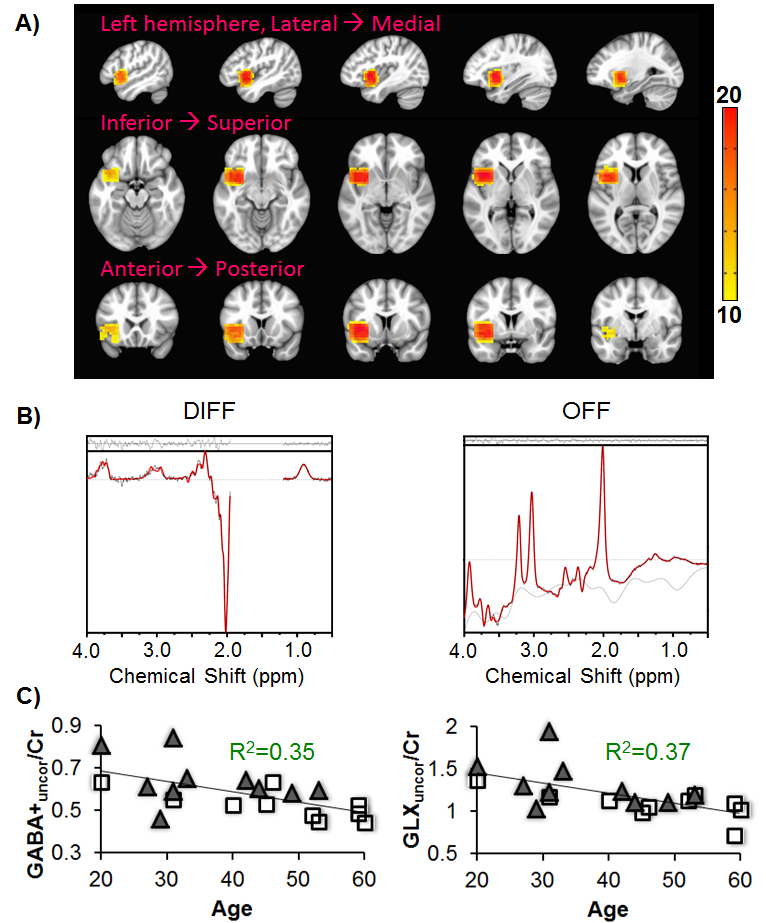


Supplementary Figure 1: (A) Average GABA MRS voxel placement in all subjects normalized to MNI space. Color bar represents number of subjects that had the GABA voxel in the spatial location. (B) Representative DIFF and OFF LC Model output from one subject. The black line is the pre-processed MR signal, and the red line is the LCModel fit. (C) The age relationship of GABA+/Cr and GLX/Cr prior to removal of age relationship. Triangles=Typical readers, Squares=Struggling readers.
